# *In vivo* performance of a tri-leaflet mechanical heart valve prosthesis in an ovine model

**DOI:** 10.1093/icvts/ivad142

**Published:** 2023-08-16

**Authors:** Tom Langenaeken, Pieter De Meester, Peter Verbrugghe, Filip Rega, Marie Lamberigts, Manon Van Hecke, Lucas Van Hoof, Bart Meuris

**Affiliations:** Department of Cardiovascular Diseases, Research Unit of Cardiac Surgery, University Hospitals Leuven, Leuven, Belgium; Congenital and Structural Cardiology, University Hospitals Leuven, Leuven, Belgium; Department of Cardiovascular Diseases, Research Unit of Cardiac Surgery, University Hospitals Leuven, Leuven, Belgium; Department of Cardiovascular Diseases, Research Unit of Cardiac Surgery, University Hospitals Leuven, Leuven, Belgium; Department of Cardiovascular Diseases, Research Unit of Cardiac Surgery, University Hospitals Leuven, Leuven, Belgium; Department of Pathology, University Hospitals Leuven, Leuven, Belgium; Department of Cardiovascular Diseases, Research Unit of Cardiac Surgery, University Hospitals Leuven, Leuven, Belgium; Department of Cardiovascular Diseases, Research Unit of Cardiac Surgery, University Hospitals Leuven, Leuven, Belgium

**Keywords:** Trileaflet mechanical valve, Sheep model, Preclinical testing, Haemodynamics, On-X

## Abstract

**OBJECTIVES:**

We present the long-term results of a trileaflet (Triflo) versus bileaflet (On-X) mechanical valve in both aortic and pulmonary positions in a sheep model.

**METHODS:**

The Triflo valve was implanted in 21 female sheep in aortic (*n* = 8) and pulmonary position (*n* = 13). The On-X valve was implanted in 7 female sheep in aortic (*n* = 1) and pulmonary (*n* = 6) positions. No antithrombotic medication of any kind was given postoperatively. In the aortic group, survival cohorts were 3 and 5 months. In the pulmonary group, survival cohorts were 10 and 20 weeks. Valve performance was assessed using haematology, echocardiography and acoustic measurements combined with post-mortem pathology analysis of the downstream organs.

**RESULTS:**

The mean gradients were lower for the Triflo valve in both pulmonary [4.30 mmHg (3.70–5.73) vs 6.80 mmHg (4.63–7.96), *P* = 0.012] and aortic [5.1 mmHg (4.2–7.7) vs 10.7 mmHg (8.7–12.9), *P* = 0.007] positions. Peak gradients were lower for the Triflo valve in both pulmonary [8.05 mmHg (6.75–10.23) vs 13.15 mmHg (9.20–14.76), *P* = 0.005] and aortic [8.7 mmHg (7.5–12.5) vs 16.5 mmHg (14.2–19.6), *P* = 0.009] positions. In both positions, leaflets and housing surface were free from any deposits macro- and microscopically and comparable to nonimplanted control valves. Peripheral organs showed no signs of thrombo-embolic damage. Biochemical and haematological were comparable to preoperative. The closing click sound pressure level of the Triflo was significantly lower in both aortic [108.4 sound pressure level (102.0–115.7) vs 111.7 sound pressure level (105.5–117.0), *P* < 0.001] and pulmonary [103.6 sound pressure level (99.1–108.9) vs 118.5 sound pressure level (116.7–120.2), *P* < 0.001] position.

**CONCLUSIONS:**

Preliminary *in vivo* results of the Triflo valve are promising in both aortic and pulmonary positions in an ovine model. Excellent haemodynamics, stable long-term function, low valve noise and no thrombo-embolic events in the absence of antithrombotic medication lay the foundation to a future clinical first-in-man trial.

**Figure:**
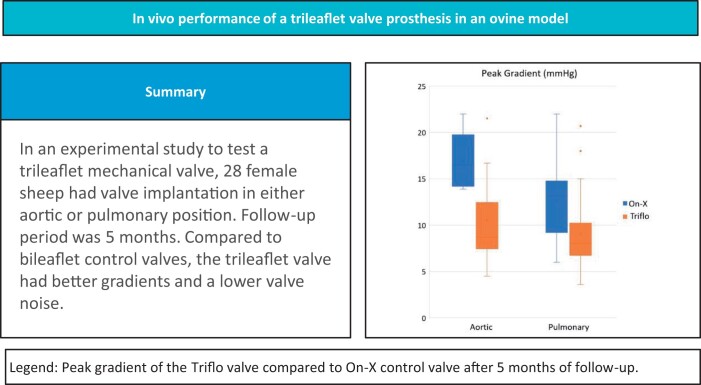

## INTRODUCTION

The quest for an ideal valve prosthesis that has lifelong durability, excellent haemodynamics and is inert to the passing blood elements remains ongoing despite intensive research. In recent long-term follow-up studies, mechanical heart valves still provide excellent durability and valve function, being absent of structural deterioration even decades after implantation [1]. Their main drawback remains the provocation of thrombosis at the surface of the valve and hinge regions, leading to valve dysfunction or distal thrombo-embolic events. The lifelong need for anticoagulation imposes an increased bleeding risk to the patient and impacts the quality of life.

Currently used mechanical heart valves all consist of a cuffed ring housing 2 leaflets, mostly made from pyrolytic carbon. The parallel leaflets of On-X valves impose a flow straightener effect, converting the normal helical flow to axial flow [2]. This leads to a drop in kinetic energy and thus total pressure loss over the valve, meaning the left ventricle experiences a higher afterload to eject the same amount of fluid into the systolic pulse [3]. This differs significantly from the Triflo valve, which conserves the physiological helical flow, angular momentum and kinetic energy [2, 4]. This closely mimics the haemodynamic profile of a natural trileaflet valve.

Currently used mechanical valves have evolved regarding design and material properties. The latest generation of On-X mechanical valves has a proven safety and efficacy at reduced INR rates [5, 6]. Therefore, the On-X valve was used as the control valve.

From previous experience, we know that the low-pressure environment of the pulmonary valve of sheep causes thrombotic changes in bileaflet mechanical valves [7]. With promising results of the Triflo valve by other authors [8–11], the aim of this study was to test the Triflo valve in the highly thrombogenic pulmonary position in a sheep model compared to the On-X valve as a control.

## MATERIALS AND METHODS

### Ethics statement

This study was approved by the Animal Ethics Committee of the University of Leuven, Leuven, Belgium (project number 189/2021).

### Study design

**Figure 1:**
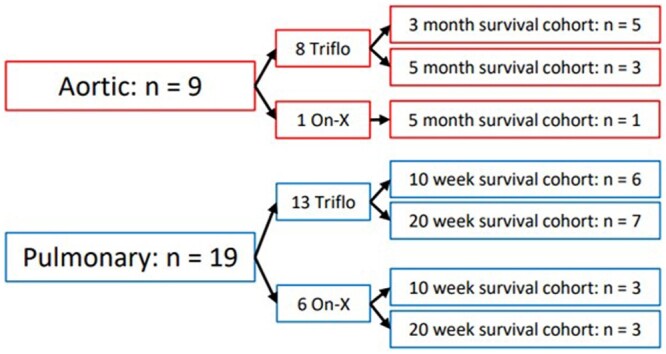
Follow-up cohorts for both groups.

A total of 28 Swifter adult sheep were acquired from the ZOOtechnical centre (ZTC, Lovenjoel, Belgium) and cared for at the animal care facility of the University of Leuven under supervision by a veterinarian in accordance with the ‘Guide for the care and use of laboratory animals, 8th Edition’, formulated by the National Research Council [12]. All animals were housed for a minimum of 1 week before valve implantation. The animals were assigned to either an aortic (*n* = 9) or pulmonary (*n* = 19) group. The follow-up cohorts were 3 and 5 months in the aortic group and 10 and 20 weeks in the pulmonary group (see Fig. 1).

### Valve

Trileaflet 21-mm mechanical valves (Triflo Heart Valve, Novostia SA) and bileaflet 21-mm mechanical valves (On-X, CryoLife and BicarbonVR, LivaNova) were used.

### Valve implantation

Animals were fasted 24 h prior to surgery. Sheep were sedated using ketamine by jugular venous injection. Arterial pressure line was inserted in the right ear and venous access line was inserted in left upper leg. Anaesthesia was induced and maintained by intravenous propophol. Positive pressure ventilation was started after intubation. A large bore gastric tube for gastric decompression was placed. Antibiotic prophylaxis by ceftiofur was given. Animals were positioned in right lateral recumbent position followed by surgical scrubbing and draping to expose the left cervical region and left chest wall.

A left anterolateral thoracotomy was made in the 3rd intercostal space. Lidocaine was given for arrhytmia prevention. The pericardium was incised anteroposteriorly and cradled by single sutures. Care was made to spare the phrenic nerve. Normothermic cardiopulmonary bypass was established by left jugular arterial and venous canulation after heparinization (heparin sodium, 250 IU/kg bodyweight; Rhone-Poulenc Rorer, Brussels, Belgium). A target ACT of 350 s reached and maintained.

In the aortic group, a left vent was placed in the left atrial appendage. After aortic cross-clamping, antegrade cold crystalloid cardioplegia is infused (500–800 ml, composition based on Plegisol, Abbott Laboratories, Chicago, IL, USA; 1.7 g bicarbonate and 120 mg lidocaine added per 1 l) followed by incision of the ascending aorta. The native valve was excised and replaced by a 21-mm Triflo or On-X in orthotopic position. The aorta was closed with a single running suture followed by cross-clamp release. Once rhythm and cardiac output was restored, the vent was removed.

In the pulmonary group, the distal pulmonary artery was clamped once sufficient venous drainage was achieved. No venting, nor cardioplegia was used. The pulmonary trunk was opened at the level of the sinotubular junction. Supplementary venous drainage was achieved by inserting a sump catheter in the right ventricle through the pulmonary valve ensuring a dry working field. The native valve was excised and a 21-mm Triflo or On-X was implanted orthotopically, in beating heart conditions. The pulmonary trunk was with a single running suture closed after properly de-airing.

In both groups, the sheep were decanulated once adequate weaning of cardiopulmonary bypass was achieved. The pericardium was left open and the chest closed in standard fashion with resorbable sutures. One pleural drain was placed. The jugular incision was closed in layers with resorbable sutures.

### Postoperative care and medication regimen

When properly awake with good parameters, animals were extubated and transferred to the intensive care unit. The chest drain was removed immediately after extubation. Feeding was allowed immediately. The first 3 postoperative days, meloxicam and buprenorphine were used for analgesia. Ceftiofur and enoxaparin (40 mg/day subcutaneously) are given the first 7 days while furosemide (1 mg/kg) is given as indicated. After the first postoperative week, all medication was stopped.

### Cardiac ultrasound

Cardiac ultrasounds for the aortic group were made monthly for up to 5 months. In the pulmonary group, cardiac ultrasound was done at 5, 10, 15 and 20 weeks. Animals were sedated allowing for good-quality imaging and measurements. The same cardiologist performed all the cardiac ultrasounds.

### Acoustic measurements

Acoustic measurements were performed at the same interval as the cardiac ultrasound using a PCB Piezotronic microphone and a NI USB-4431 data acquisition system, capturing sound at a 96-kHz rate with 24-bit resolution. The measurements were digitally band-pass filtered using a fourth-order Butterworth filter with lower and upper cut-off frequencies set to 1 and 16 kHz, respectively, to focus on the frequency range dominated by the valve prosthesis sound. Subsequently, the peak root-mean-square sound pressure level was determined for each measured cardiac cycle, using an integration time of 3.75 ms. Closing clicks were automatically detected.

### Explant procedures

After completion of their designated follow-up period, animals were euthanized with pentobarbital after heparinization (300 IU/kg) to prevent post-mortem clotting. A cardiectomy was performed. The valves were carefully dissected from the surrounding tissue and assessed for evidence of structural degradation, thrombus (weight), vegetation, pannus overgrowth, hinge integrity and paravalvular leak. In the pulmonary group, the lungs are macroscopically evaluated and random lung biopsies were taken to screen for thrombo-embolism. In the aortic group, the downstream organs (brains, liver, een, kidneys) were macroscopically evaluated and random biopsies were taken to screen for thrombo-embolism. Macroscopic photographs of the explanted valves were taken.

### Data analysis

Study outcome data by valve type and position were described using mean and standard deviation or median and interquartile range for continuous variables, depending on the normality of distribution assessed with the Shapiro–Wilk test. Univariable analysis was unpaired Student’s *t*-tests for normally distributed variables and Mann–Whitney *U* tests for not normally distributed variables. A significance level of *P* < 0.05 was used. All tests were 2 sided. Software used was IBM SPSS Statistics Software (IBM Corporation, NY, USA).

## RESULTS

### Perioperative outcomes and survival

All sheep survived until study termination. There was no bleeding or distal thrombo-embolic events. There were no signs of cardiac decompensation or infection.

### Cardiac ultrasound

**Figure 2:**
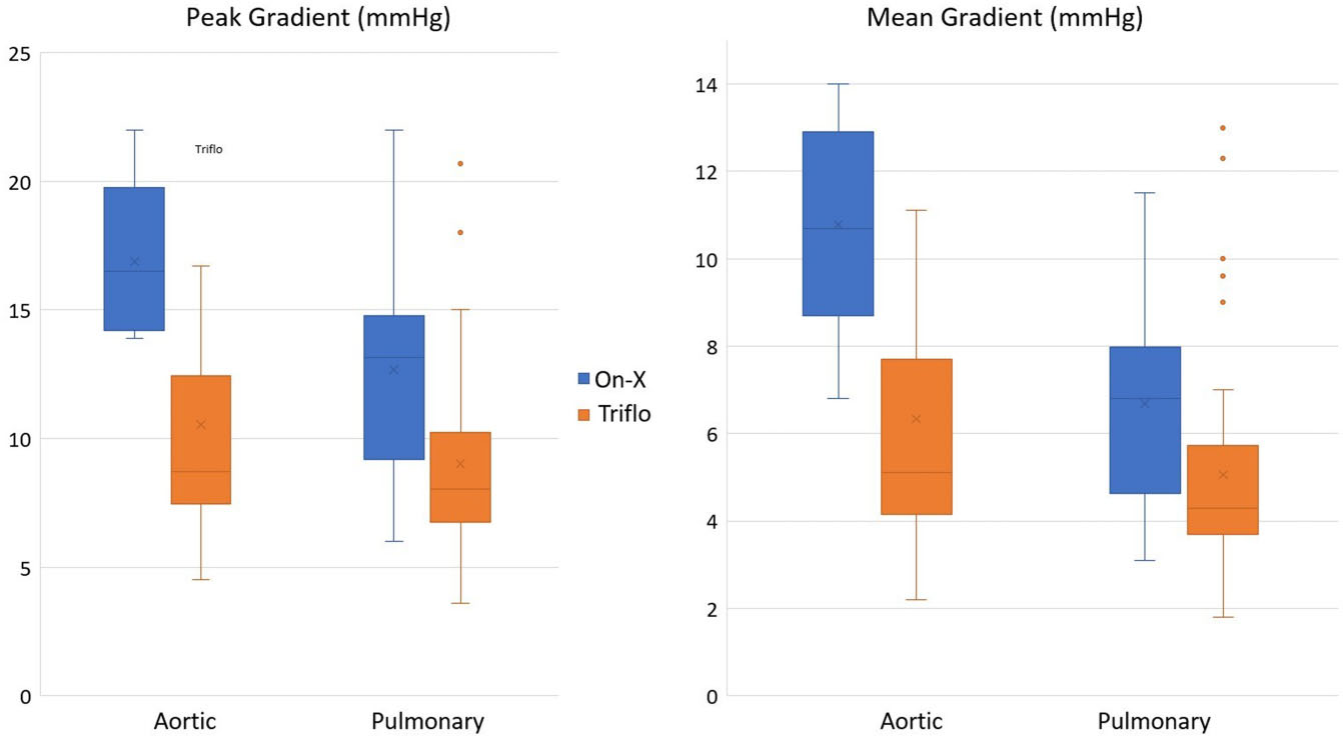
Clustered box plot diagram for peak and mean gradients in both positions.

The Triflo has significantly better mean and peak gradients in both aortic and pulmonary positions (see Table 1 and Fig. 2). While the effective orifice area tends to be higher for the Triflo, this difference was not significant at an alpha level of 0.05. The cardiac output was comparable between groups, confirming that the differences in valve parameters are truly valvular in origin, and not due to cardiac performance differences in both groups.

**Table 1:** Cardiac ultrasound results for both valve types in both positions

	Trileaflet	On-X	*P*-Value
Aortic			
Mean gradient (mmHg)	***5.1 (4.2–7.7)***	***10.7 (8.7–12.9)***	***0.007***
Peak gradient (mmHg)	***8.7 (7.5–12.5)***	***16.5 (14.2–19.6)***	***0.009***
Effective orifice area (cm²)	2.06 ± 0.28	1.72 ± 0.48	0.186
Cardiac output (l/min)	6.02 ± 1.62	5.76 ± 0.99	0.681
Pulmonary			
Mean gradient (mmHg)	***4,30 (3.70–5.73)***	***6.80 (4.63–7.96)***	***0.012***
Peak gradient (mmHg)	***8.05 (6.75–10.23)***	***13.15 (9.20–14.76)***	***0.005***
Effective orifice area (cm²)	2.00 (1.50–2.40)	1.91 (1.40–2.70)	0.246
Cardiac output (l/min)	5.06 ± 1.09	5.54 ± 1.28	0.183

Peak and mean gradient: mean and standard deviation—unpaired Student's *t*-test. Effective orifice area and cardiac output: median and IQR—Mann–Whitney *U*-test. Statistically significant values are in italics.

IQR: interquartile range.

### Acoustic measurements

**Figure 3:**
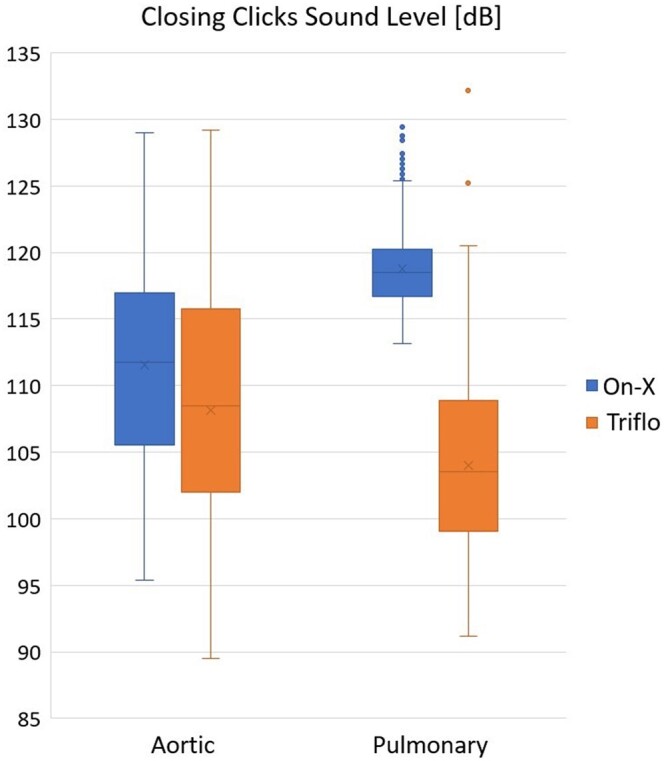
Clustered box plot for closing click sound level in both positions. SPL: sound pressure level.

Acoustic results indicate that the Triflo valve is significantly more quiet than the On-X valve (see Table 2 and Fig. 3).

**Table 2:** Closing click volume of both valve types (median and IQR)

Closing click volume (SPL)	Trileaflet	On-X	*P*-Value
Aortic	***108.4 (102.0–115.7)***	***111.7 (105.5–117.0)***	***<0.001***
Pulmonary	***103.6 (99.1–108.9)***	***118.5 (116.7–120.2)***	***<0.001***

Mann–Whitney *U*-test was used.

IQR: interquartile range; SPL: sound pressure level.

### Macroscopic valve assessment

**Figure 4:**
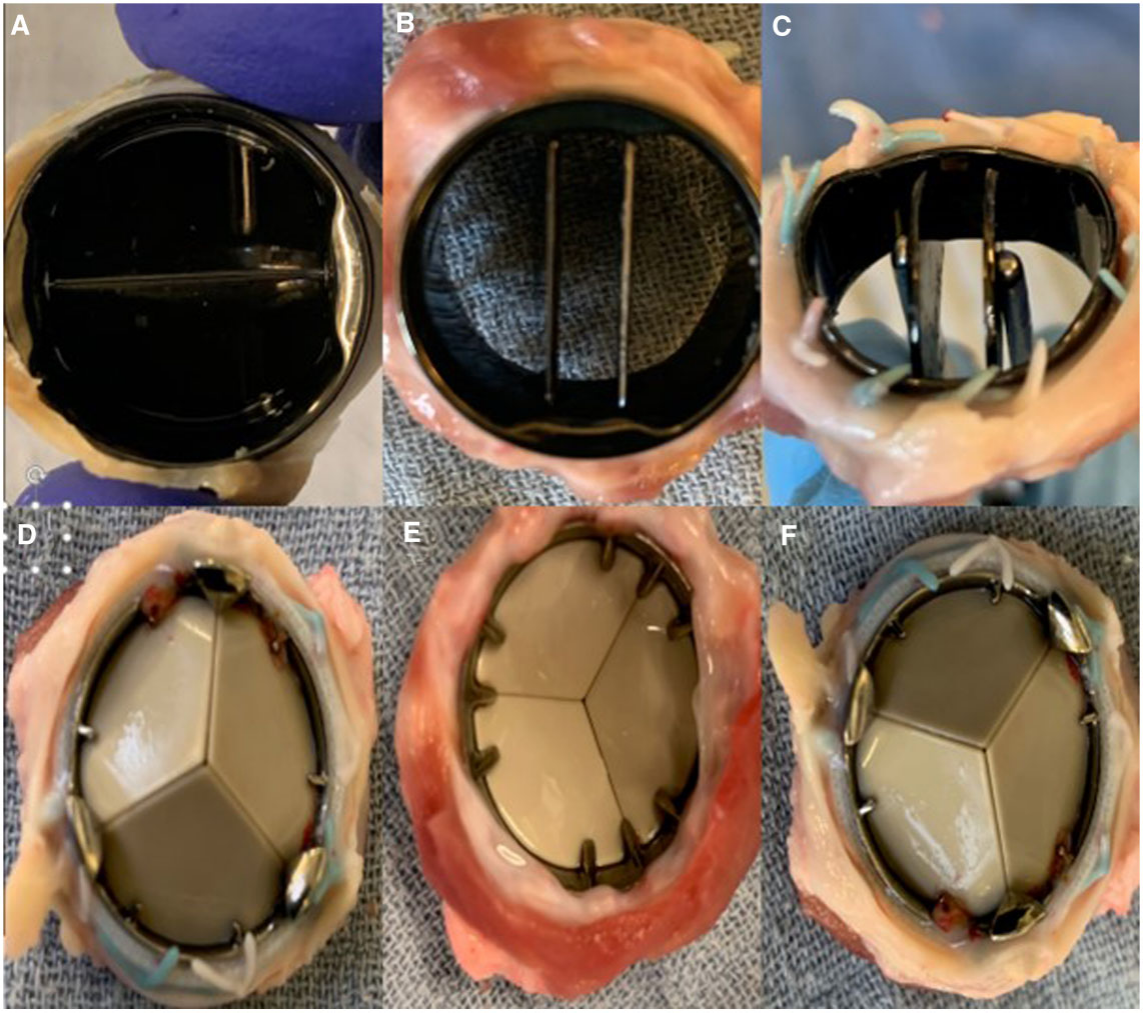
On-X valve (**A**–**C**) and Triflo valve (**D**–**F**) in pulmonary position after 10 weeks.

**Figure 5:**
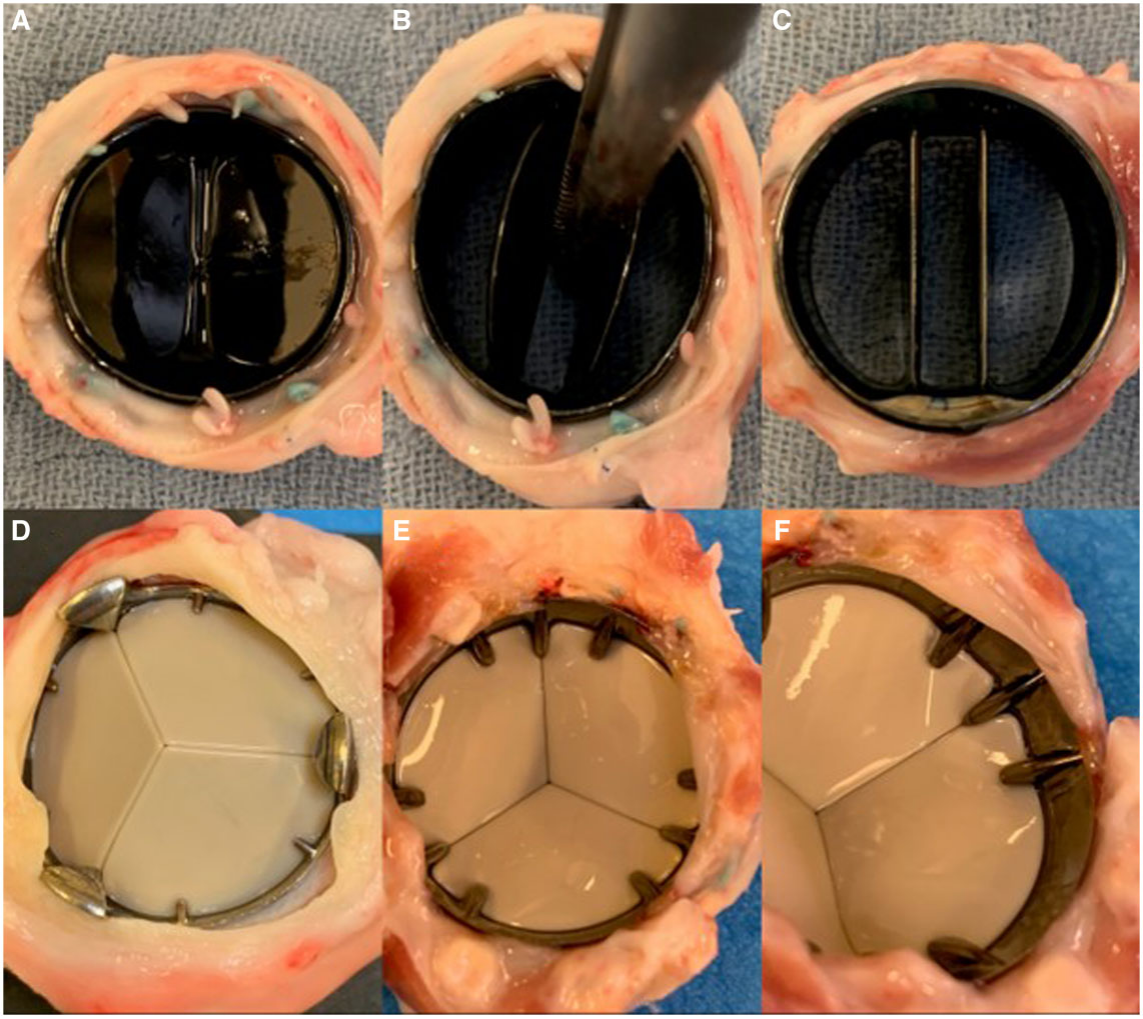
On-X valve (**A**–**C**) and Triflo valve (**D**–**F**) in pulmonary position after 20 weeks.

**Figure 6:**
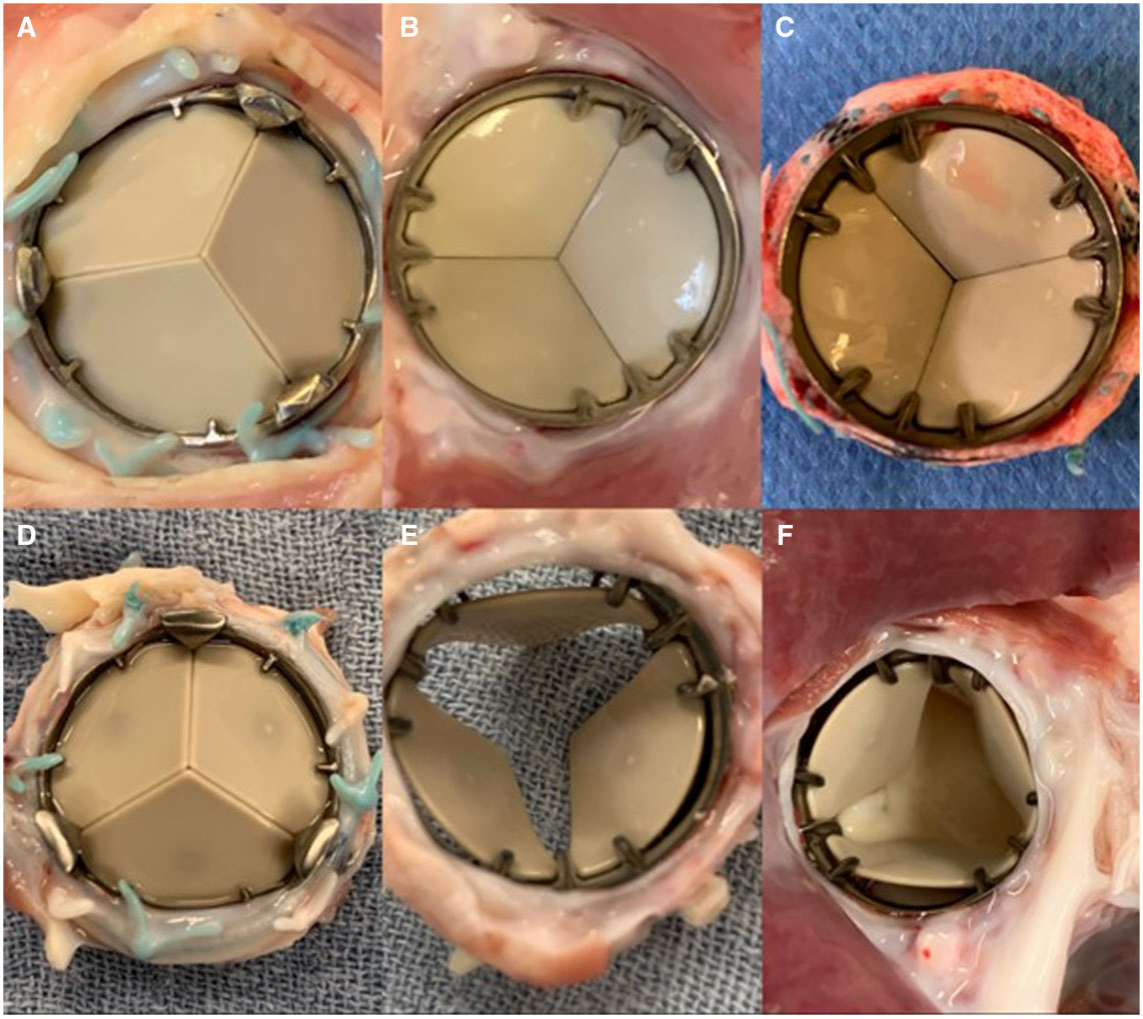
Triflo valve in aortic position at 3 months (**A**–**C**) and 5 months (**D**–**F**).

After animal sacrifice, a full necropsy was done on all animals. No macroscopic signs of thrombo-embolic disease or bleeding were detected. All annuli were perfectly healed. The sewing cuffs were enclosed by dense connective tissue without overlap on the valve ring or obstruction of the leaflets. Inspection of the explanted valves showed clean valvular surfaces without any form of thrombosis in both positions for both survival cohorts (see Figs 4–6). The hinge mechanisms in both valve types maintained normal hinge mobility in both aortic and pulmonary positions.

### Microscopic valve assessment

Random biopsies of the lungs in the pulmonary group and the various downstream organs of the aortic group (brain, liver, spleen, kidneys) were absent of microscopic signs of thrombo-embolic disease. No signs of infection were present.

### Blood results

Laboratory values were absent for ongoing infection or signs of haemolysis due to valve implantation. No other observable effects of valve implantation could be found.

## DISCUSSION

The quest for a cardiac valve prosthesis that is durable, inert to the passing blood elements and allows that good haemodynamics is still ongoing. While mechanical valves are durable and can provide excellent outcomes in the long term, lifelong anticoagulation remains paramount [13, 14]. The biologic valves do not require anticoagulation but their lifespan is limited, limiting their use in the younger population due to the risk of structural valve degeneration requiring reintervention [1]. While the currently recommended age threshold for opting for a mechanical heart valve over, a biological is 60–65 years old; many younger patients still prefer a biological valve to avoid the lifelong coagulation need and associated burden [15–18]. There is an unmet need for a valve that combines the durability of a mechanical valve with the blood compatibility of a biological valve.

Several authors have tested a Triflo valve before. Lapeyre *et al.* [9] were the first in 1994, implanting a Triflo in mitral position in 6 calves. Long-term survival in 4 calves was achieved, and 2 suffered from non-valvular premature death. Anticoagulation was given to all calves in the form of heparin to maintain a PTT at 1.5–2.0× baseline for 1 week. Two calves were kept fully anticoagulated (orally administered sodium warfarin), in 1 calve anticoagulation was stopped after 1 month and in the final calve no anticoagulation was given after 1 week but antiplatelet therapy was started in the 5th month and continued until the study termination at 9 months. Their results were already promising with stable valve function, acceptable gradients despite tripling of the animal body weight and no signs of haemolysis or distal thrombo-embolic events. Sato *et al.* [11] followed in 2003 with a series of 8 calves implanted with a Triflo type IIA in mitral position. Six animals completed the follow-up period of 5 months, during which no anticoagulation of any kind was given. Valves were clean microscopically at explantation and no emboli were observed but in 1 case.

The first comparison study of the Triflo valve was authored by Gregoric *et al.* [10] in 2004. 6 calves were implanted with a Triflo valve in aortic position and compared with 3 calves with a St. Jude Medical valve in aortic position. No anticoagulation or antiplatelet medication of any kind was given. At a follow-up duration of 159 days, the Triflo valve showed excellent if not superior haemodynamics than the St. Jude Medical valve, which suffered from high transvalvular gradients and left ventricular hypertrophy.

The first study using sheep was performed by Gallegos *et al.* [8] in 2006. A total of 26 sheep were implanted with the Triflo valve in either aortic (*n* = 19, 21 mm) or mitral (*n* = 8, 29 mm) position. Again, no anticoagulation or antiplatelet drugs of any kind were given. The follow-up period was 150 and 365 days. It was concluded that the valve performed equally if not superior to the St. Jude Medical valve with regards to overall haemodynamics and myocardial hypertrophy.

The good haemodynamics already proven in the previous animal models could be confirmed in this study. The Triflo valve was designed to maximally mimic the native trileaflet aortic valve, which allows a central undisturbed flow with minimal turbulence and maximal orifice area. When compared to a bileaflet valve of equal size, the Triflo has a tendency towards a greater orifice area. The EAO measured at the different intervals almost reached 2.0 which is better than most biological valves. Combined with the low-pressure gradients (significantly lower than the On-X), this can predict good left ventricle function, exercise capacity and survival [13, 19–21]. It also reduces the risk of patient-prosthesis mismatch and thus reduces all-cause and cardiac-related mortality [22].

Another interest in this study was thrombogenicity. Valve thrombosis in an experimental setting in pulmonary position in sheep has been described before, due to its low-pressure environment [7]. The most striking result of this study is the total absence of any kind of thrombus on the valve leaflets, struts or annular ring for both valve types. The downstream organs were absent of device-related macro- or microscopic changes. This is promising and can be of value for future patients who can benefit from lower or even no anticoagulation. This study also confirms that the Triflo is a quiet valve, inaudible in ambient noise whereas the classic On-X valve of pyrolytic carbon is clearly audible. This is not only due to the use of poly(ether-ether-ketone) for the leaflets, but also due to a slower and thus more controlled opening and closure of the valve. This feature should not be underestimated, as up to 14.2% of mechanical valve patients suffer from valve sound-related complaints [23].

### Limitations

Sheep are considered the gold standard for preclinical evaluation of (mechanical) heart valve prosthesis. However, there are concerns of insufficient thrombogenicity of these animals. Most notorious is the clinical discontinuation of the Medtronic Parallel valve due to unusually high rates of thrombosis in patients despite good preclinical results in sheep [24]. The same concerns have been raised for bovine models. Porcine models seem the better alternative due to a coagulation system that is very similar to humans, with some authors even stating them as hypercoagulable, especially in the immediate postoperative phase [25–27]. However, the main goal of this study was a safety assessment of the Triflo valve. Statements regarding thrombogenicity of this new valve have to be interpreted with caution. Further studies in even more thrombogenic situations have to be planned. A study analysing the Triflo valve in pulmonary position in a porcine model is pending.

## CONCLUSION

The Triflo valve reveals excellent function and safety in both aortic and pulmonary positions. Excellent haemodynamics, stable long-term function, low valve noise and no thrombo-embolic events in the absence of antithrombotic medication lay the foundation to a future clinical first-in-man trial.

## Data Availability

Data underlying this article will be shared upon reasonable request to the corresponding author.
